# An Overlapping Case of IgG4-Related Disease and Systemic Lupus Erythematosus

**DOI:** 10.1177/2324709619862297

**Published:** 2019-07-18

**Authors:** Srikanth Naramala, Sharmi Biswas, Sreedhar Adapa, Vijay Gayam, Venu Madhav Konala, Subhasish Bose

**Affiliations:** 1 Adventist Medical Center Hanford, Hanford, CA, USA; 2 Weill Cornell Medicine, New York, NY, USA; 3 Kaweah Delta Medical Center, Visalia, CA, USA; 4 Interfaith Medical Center, Brooklyn, NY, USA; 5 Ashland-Bellefonte Cancer Center, Ashland, KY, USA; 6 University of Virginia, Charlottesville, VA, USA

**Keywords:** lupus, systemic lupus erythematosus, SLE, IgG4-related disease

## Abstract

We are reporting a case of a 63-year-old Chinese female who presented to the rheumatology clinic with positive antinuclear antibody and unintentional weight loss along with lymphadenopathy. Further workup revealed eosinophilia, elevated anti–double stranded DNA, serum protein, and serum IgG4 (immunoglobulin G4). The patient was diagnosed with systemic lupus erythematosus. Due to the raised IgG4 level along with eosinophilia and diffuse lymphadenopathy, IgG4-related systemic disease was suspected. It was confirmed with IgG4 staining on lymph node biopsy. Our case is presenting the fact that systemic lupus erythematosus and IgG4-related disease can be present in the same patient with multiple overlapping features making accurate diagnosis challenging.

## Introduction

Systemic lupus erythematosus (SLE) is a chronic autoimmune disease with a variable clinical presentation and can affect any part of the body. IgG4 (immunoglobulin G4)-related disease (IgG4-RD) is a systemic fibroinflammatory disease with protean manifestations involving virtually any organ in the body. Hallmarks of IgG4-related disease are lymphoplasmacytic tissue infiltration, fibrosis (often in storiform pattern), obliterative phlebitis, and elevated serum IgG4 concentration. Treatment mainly involves use of steroids and immunosuppressive agents. We are reporting a case of a 63-year-old female presenting with joint pains, fatigue, unintentional weight loss along with lymphadenopathy with an unusual overlap of SLE and IgG4-RD.

## Case Presentation

A 63-year-old Chinese female presented to rheumatology clinic with positive antinuclear antibody 1:80 homogeneous pattern, severe fatigue, hair loss, joint pains for approximately last 3 years, unintentional weight loss of 24 pounds (20% of her ideal body weight) in last 8 months, and lymphadenopathy. Her joint pains are localized to bilateral hands, elbows, shoulders, hips, and knees. Of all the joints her hands hurt her the most. Joint pains are associated with intermittent swelling and early morning stiffness lasting at least for 30 minutes. Her symptoms were worse during winter and cold weather. She is unable to do her activities of daily living like holding a coffee mug, eating with a spoon, opening bottles, and so on.

She was evaluated for underlying malignancy in the setting of generalized lymphadenopathy and significant unintentional weight loss. Her past medical history was significant for hypertension, thyroid nodule status post ultrasound-guided fine needle aspiration cytology consistent with benign follicular colloidal nodule and bilateral carpal tunnel syndrome on electromyography status post nerve release.

Her medications include benazepril-hydrochlorothiazide 20 mg–12.5 mg, diclofenac potassium 50 mg PRN, vitamin D_3_ 2000 international units. Family history was significant for cancer in paternal grandmother and hypertension and hypercholesteremia in mother. She is an ex-smoker with 30 pack-year smoking history and quit smoking 2 years ago.

Physical examination was positive for thin, cachectic female with palpable posterior cervical, supraclavicular, and bilateral axillary lymphadenopathy. Tenderness was felt in multiple proximal inter phalangeal joints of bilateral hands with mild synovitis. Diffuse thinning of hair on scalp was noted.

Further review of records showed intermittent eosinophilia on the complete blood count. Subsequent work-up showed elevated anti–double stranded DNA 1:40 by immunofluorescence assay (normal <1:10), elevated IgG4 level of 452 mg/dL (normal = 1-123 mg/dL), elevated serum protein 9.7 g/dL, serum globulin 7.1 g/dL, serum protein electrophoresis showing polyclonal increase in the gamma region with no M-spike, and erythrocyte sedimentation rate of 67 mm/h.

Other testing was negative for smith antibody, ribonuclear protein antibody, anti-SSA, anti-SSB, rheumatoid factor, anti-cyclic citrullinated peptide, anti-phospholipid antibody panel, anti-neutrophilic cytoplasmic antibody with myeloperoxidase, and proteinase 3 antibody. C3 and C4 levels within normal limits. Urinalysis and renal function were normal. Infection workup was negative for hepatitis, HIV, tuberculosis (quantiferon gold), syphilis (rapid plasma reagin), and coccidiomycosis. Peripheral blood flow cytometry showed no flow cytometric evidence of monoclonality, acute leukemia, or lymphoproliferative disorder.

**Figure 1.**
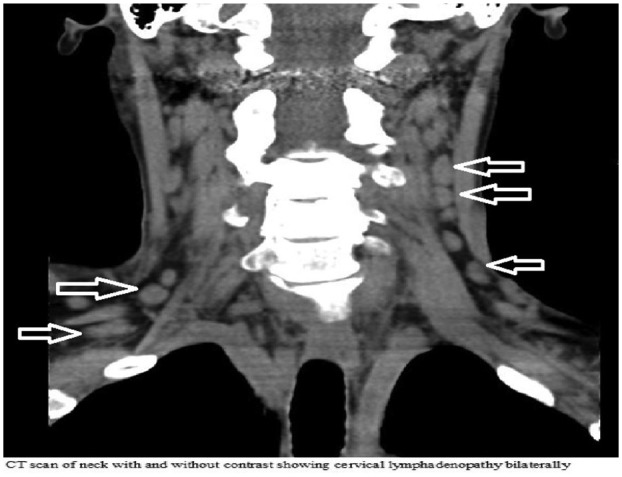
CT scan neck with and without contrast showing bilateral cervical adenopathy.

A computed tomography scan of the neck, chest, abdomen, and pelvis with and without contrast showed prominent cervical (Figure 1), axillary lymphadenopathy along with multiple shotty lymph nodes in the region of the mediastinum and left periaortic region. Other significant finding was bilateral pleural effusions.

Age-specific cancer screening was negative for malignancy including pap smear, colonoscopy, and bilateral screening mammogram.

**Figure 2.**
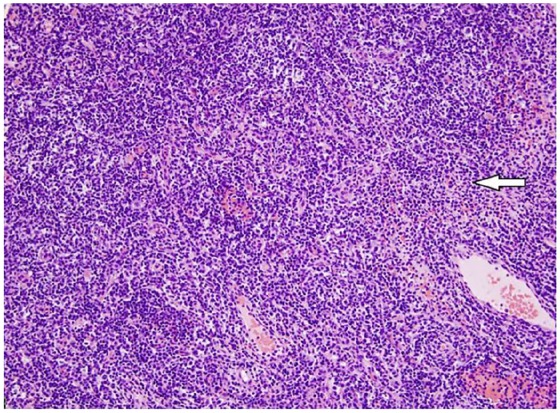
Low-power field (20×; hematoxylin-eosin) with arrow pointing to rich lymphoplasmacytic infiltrate on axillary lymph node biopsy.

**Figure 3.**
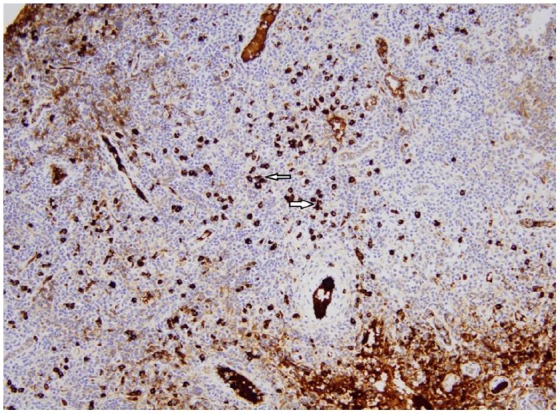
Low-power field (20×) showing abundant IgG4 plasma cells on IgG4 staining. Arrows pointing to IgG4-positive plasma cells.

Ultrasound-guided fine needle aspiration cytology of bilateral axillary lymph nodes was negative for malignancy with abundant plasma cells and positive IgG4 staining (Figures 2 and 3).

She was diagnosed with overlapping syndrome of IgG4 related disease and SLE. She was started on 10 mg prednisone and 200 mg plaquenil daily, which significantly improved her symptoms. Her prednisone was tapered slowly and completely discontinued in 5 months. She is currently on maintenance plaquenil 200 mg daily back to her usual state. She gained weight, and her other symptoms of fatigue, hair loss, joint pains, and lymphadenopathy completely resolved. Her repeat anti–double stranded DNA, IgG4, and eosinophil levels were within normal limits (Table 1).

**Table 1.** IgG4 and Anti-dsDNA From Diagnosis and Further Follow-up.

	Initial Diagnosis	2 Months After Treatment	8 Months After Treatment	12 Months After Treatment
IgG4 (1-123 mg/dL)	452	245	71	68
Anti-dsDNA (normal <1:10)	1:40	<1:10	<1:10	<1:10

Abbreviations: IgG4, immunoglobulin G4, anti-dsDNA, anti–double stranded DNA.

## Discussion

SLE is a chronic autoimmune disease with a variable clinical presentation and can affect any part of the body. The most common presentation is a mixture of symptoms affecting the skin, musculoskeletal system, hematologic, and fatigue along with positive serologies. The diagnosis of SLE should be based on a combination of clinical and immunologic findings as described in Table 2.^1^

**Table 2.** 2012 Systemic Lupus International Collaborating Clinics (SLICC) Criteria for SLE.

A. Joints	1. Synovitis in 2 or more joints associated with tenderness, swelling, and morning stiffness for at least 30 minutes
B. Skin	1. Oral ulcers 2. Chronic cutaneous lupus 3. Acute cutaneous lupus or subacute cutaneous lupus 4. Nonscarring alopecia
C. Hematologic	1. Thrombocytopenia (<100 000/mm^3^) at least once 2. Leukopenia (<4000/mm^3^ at least once) or lymphopenia (<1000/mm^3^ at least once) 3. Hemolytic anemia
D. Serositis	1. (a) Pericardial pain > 1 day or pericarditis by electrocardiography or pericardial effusion or pericardial rub (b) Pleurisy > 1 day or pleural rub or pleural effusions
E. Renal	1. Urine protein-to-creatinine ratio (or 24-hour urine protein) representing 500 mg protein/24 hours or red blood cell casts
F. Immunologic	1. Positive ANA level 2. Positive anti-Smith antibody level 3. Positive anti-dsDNA antibody level 4. Low complement—low C3 or low C4 or low CH50 5. Direct Coombs test in the absence of hemolytic anemia 6. Positive antiphospholipid antibody testing as determined by any of the following: positive lupus anticoagulant, anticardiolipin antibody level, anti-β_2_-glycoprotein I or false-positive test result for rapid plasma regain
G. Diagnosis	1. Patient can be classified as having SLE if he or she satisfies 4 with at least 1 clinical and 1 immunologic criteria used in the SLICC criteria, OR if he or she has biopsy-proven lupus nephritis.

Abbreviations: SLE, systemic lupus erythematosus; ANA, antinuclear antibody; anti-dsDNA, anti–double stranded DNA.

Our patient satisfied 4 criteria with at least 1 clinical and 1 immunologic criteria as mentioned in the case presentation and was labelled as SLE.

IgG4-RD is a systemic fibroinflammatory disease with protean manifestations involving virtually any organ in the body. Hallmark of IgG4-RDs are lymphoplasmacytic tissue infiltration, fibrosis (often in storiform pattern), obliterative phlebitis, and elevated serum IgG4 concentration. Most commonly seen in middle-aged and older people; mean age from 59 to 68 years and more common in men (70% to 80%).^2,3^ Serum IgG4 levels are elevated in approximately 60% of patients. Up to 40% of patients with IgG4-RD have a peripheral eosinophilia and elevated IgE levels.^2,3^ Our patient had elevation of IgG4 level and peripheral blood eosinophilia. Her IgE level was not checked.

IgG4 is an antibody that has a very unique structure and function. It is the least abundant antibody and comprises less than 5% of antibody in human class. Generally, IgG4 level in human body remains always stable in normal condition.

Autoimmunity is one of the commonest triggers for IgG4-RD with key role of T helper-2 cell (Th2) cell involvement in the pathophysiology. Th2 cells overexpress interleukins 4, 5, 10, and 13 as well as transforming growth factor-β, which resulted in increased eosinophils as well elevated IgG4 and IgE concentrations. It also contributes to activation of fibroblasts leading to fibrosis.^4^

IgG4-related systemic disease can involve pancreas, biliary tree, salivary glands, periorbital tissues, kidneys, lungs, lymph nodes, meninges, aorta, prostate, breast, thyroid gland, pericardium, and skin.

The diagnostic approach of IgG4-related systemic disease should be based on a combination of clinical evaluation, laboratory parameters, imaging, and biopsy results. Initially proposed criteria were centered on specific organs rather than systemic IgG4-RD. In 2011, Umehara et al^5^ proposed a set of criteria for the diagnosis of systemic IgG4-RD designed to be used independently of the predominant organ: (1) serum IgG4 concentration >135 mg/dL and (2) >40% of IgG+ plasma cells being IgG4+ and >10 cells/high-powered field of biopsy sample. Validation of these criteria needs to be tested further by conducting international multidisciplinary collaboration with an expert panel. Our patient had elevated serum IgG4 level along with abundant IgG4-positive plasma cells on the biopsy.

Sarles et al^6^ first described a form of IgG4-RD as sclerosing pancreatitis in 1961. It was later termed as autoimmune pancreatitis. Since then, IgG4-RD has been described in nearly every organ system. Enlargement of organ is the key finding for clinical suspicion of IgG4-RD. Organomegaly is detected either through physical examination or in incidental finding on diagnostic imaging.^4,7^ Symptoms related to specific organs can be also the primary features like abdominal pain, respiratory symptoms, diarrhea, and pruritis.^4,7^ Lymphadenopathy is seen in 25% patients approximately.^3,4^ In laboratory tests, mild to moderate eosinophilia is present in one third patients.^4^ Computed tomography and magnetic resonance imaging findings are very important to support the diagnosis of IgG4-RD. Due to heterogeneous characteristics of IgG4-RD signs and symptoms, diagnosis is very challenging.

The main therapeutic option in IgG4-RD is glucocorticoids. Based on disease presentation, immunosuppressive drugs are also being used. In 90% reported cases, azathioprine was used as an immunosuppressive agent. Anecdotal data and multiple case reports mention the use of other immunosuppressive agents such as cyclophosphamide methotrexate, mycophenolate mofetil, and tacrolimus. Biologic agents (Rituximab) and surgery are also available options to treat depending on the symptoms and clinical findings. Because of rarity of the disease, no randomized controlled trails have been conducted and most of the treatment options are derived from individual experience, case reports, and case series.^8^

## Conclusion

We report an unusual case of overlap of SLE without any kidney involvement and IgG4-RD. Review of the literature shows only one case describing an overlap of IgG4-RD and lupus nephritis. Our case highlights that these 2 diseases can overlap in some patients, which was never reported in the past.
